# Cortical Dysfunction Underlies the Development of the Split-Hand in Amyotrophic Lateral Sclerosis

**DOI:** 10.1371/journal.pone.0087124

**Published:** 2014-01-24

**Authors:** Parvathi Menon, Matthew C. Kiernan, Steve Vucic

**Affiliations:** 1 Sydney Medical School Westmead, University of Sydney, Sydney, NSW, Australia; 2 Neuroscience Research Australia, Sydney, NSW, Australia; 3 Brain and Mind Research Institute, University of Sydney, Sydney, NSW, Australia; University of Florida, United States of America

## Abstract

The split-hand phenomenon, a specific feature of amyotrophic lateral sclerosis (ALS), refers to preferential wasting of abductor pollicis brevis (APB) and first dorsal interosseous (FDI) with relative preservation of abductor digiti minimi (ADM). The pathophysiological mechanisms underlying the split-hand phenomenon remain elusive and resolution of this issue would provide unique insights into ALS pathophysiology. Consequently, the present study dissected out the relative contribution of cortical and peripheral processes in development of the split-hand phenomenon in ALS. Cortical and axonal excitability studies were undertaken on 26 ALS patients, with motor responses recorded over the APB, FDI and ADM muscles. Results were compared to 21 controls. Short interval intracortical inhibition (SICI), a biomarker of cortical excitability, was significantly reduced across the range of intrinsic hand muscles (APB_SICI ALS_ 0.3±2.0%, APB_SICI controls_ 16.0±1.9%, P<0.0001; FDI_SICI ALS_ 2.7±1.7%, FDI _SICI controls_ 14.8±1.9%, P<0.0001; ADM_SICI ALS_ 2.6±1.5%, ADM _SICI controls_ 9.7±2.2%, P<0.001), although the reduction was most prominent when recorded over APB/FDI. Changes in SICI were accompanied by a significant increase in motor evoked potential amplitude and reduction of cortical silent period duration, all indicative of cortical hyperexcitability, and these were most prominent from the APB/FDI. At a peripheral level, a significant increase in strength-duration time constant and reduction in depolarising threshold electrotonus were evident in ALS, although these changes did not follow a split-hand distribution. Cortical dysfunction contributed to development of the split-hand in ALS, thereby implying an importance of cortical hyperexcitability in ALS pathogenesis.

## Introduction

Amyotrophic lateral sclerosis (ALS) is a rapidly progressive neurodegenerative disorder of the motor neurons[1]. Weakness and wasting of the abductor pollicis brevis (APB) and first dorsal interosseous (FDI) muscles, with relative preservation of abductor digiti minimi (ADM), may be a clinical feature of ALS, termed the ***split-hand*** [2], [3]. Although the pathophysiological mechanisms underlying the development of this split-hand has not been established, central and peripheral processes have been implicated [4], [5], [6]. Resolution of this issue could provide unique insights into ALS pathophysiology and potentially guide future neuroprotective strategies.

Debate continues regarding the mechanisms of motor neuron degeneration in ALS. It has been argued that corticomotoneuronal hyperexcitability may induce anterior horn cell degeneration via an anterograde glutaminergic mechanism [7]. Support for such a mechanism has been indirectly provided by transcranial magnetic stimulation (TMS) studies establishing cortical hyperexcitability as an early feature of ALS and linked to neurodegeneration[8], [9], [10], [11], [12]. Given that APB and FDI muscles are critical for execution of complex hand tasks, and thereby exhibit a larger cortical representation [13], it could follow that preferential dysfunction of corticomotoneuronal pathways innervating the APB/FDI motor neurons may underlie development of the split-hand [4].

Previous TMS studies have established significant differences in cortical excitability across a range of upper limb muscles in healthy subjects[14], [15]. Specifically, the degree of intracortical inhibition and corticomotoneuronal output was greater to thenar muscles compared to biceps brachii and hypothenar muscles, suggesting a greater cortical representation and corticomotoneuronal projections to thenar muscles [14], [15]. Interestingly, preferential dysfunction of thenar corticomotoneuronal projections has been reported in ALS [16].

Peripheral processes have also been implicated in development of the split-hand phenomenon. Studies in healthy controls have reported more prominent persistent Na^+^ conductances and less K^+^ currents in the APB and FDI motor axons [5], thereby suggesting that motor axons innervating the APB/FDI were hyperexcitable and prone to degeneration. Underscoring this notion were findings of more prominent hyperexcitability of APB axons in a Japanese ALS cohort [17]. In contrast, a more recent study established that axonal dysfunction was evident across the range of intrinsic hand muscles and was not consistent with a split-hand distribution [18]. Rather, it was suggested that abnormalities of axonal excitability may reflect downstream effects of primary neurodegenerative processes [18]. Consequently, the aim of the present study was to determine whether cortical hyperexcitability underlies the development of the split-hand sign in ALS.

## Materials and Methods

Cortical and axonal excitability studies were undertaken on 26 patients with clinically probable or definite ALS as defined by the Awaji criteria [19]. The diagnosis of ALS was confirmed in patients initially classified as probable ALS by longitudinal follow-up. All patients provided written informed consent to the procedures which were approved by the Western Sydney Local Health District Human Research Ethics Committee.

### Clinical phenotype

All ALS patients were clinically staged using the amyotrophic lateral sclerosis functional rating scale-revised (ALSFRS-R) score [20] and categorised according to site of disease onset. Muscle strength was assessed using the Medical Research Council (MRC) score [21], with the following muscle groups assessed bilaterally yielding a total MRC score of 90: shoulder abduction; elbow flexion; elbow extension; wrist dorsiflexion; finger abduction; thumb abduction; hip flexion; knee extension; ankle dorsiflexion.

### Cortical excitability

Cortical excitability studies were undertaken by applying a 90 mm circular coil connected to two high-power magnetic stimulators connected via a BiStim device (Magstim Co., Whitlands, South West Wales, UK) with recording of motor evoked response over the APB FDI and ADM muscles. The circular coil was chosen over a focal (figure-of-eight) coil as the former was easier to use with less frequent overheating of the coil itself. Importantly, previous studies reported no qualitative differences in the pattern of inhibition and facilitation when using either a circular coil or a focal (figure-of-eight) coil [14]. In addition, a previous study incorporating the threshold tracking TMS technique utilised a focal coil [22] and established a similar pattern of. This study reported two phases of short-intracortical inhibition, ISI≤ms and at 3 ms, an identical pattern of short-interval intracortical inhibition to that reported in our own normative study [23].

The circular coil was adjusted in both antero-posterior and medial-lateral direction until the optimal position for an MEP response was obtained from the relevant muscle according to a previously reported technique [24]. Specifically, the optimal scalp position was established by determining the site at which the smallest TMS stimulus intensity (threshold) evoked an MEP response. This point on the scalp was marked with a skin marking pencil and the coil was positioned over this site for the duration of the experiment by a purpose built coil stand.

**Paired-pulse threshold tracking TMS** was undertaken according to a previously reported technique [23]. Briefly, the MEP amplitude was fixed and changes in the test stimulus intensity required to generate a target response of 0.2 mV (±20%), when preceded by sub-threshold conditioning stimulus, was measured. Resting motor threshold (RMT) was defined as the stimulus intensity required to maintain the target MEP response of 0.2 mV (±20%).

Short-interval intracortical inhibition (SICI) was determined over the following interstimulus intervals (ISIs): 1, 1.5, 2, 2.5, 3, 3.5, 4, 5, and 7 ms, while intracortical facilitation (ICF) was measured at ISIs of 10, 15, 20, 25 and 30 ms. Stimuli were delivered sequentially as a series of three channels: **channel 1**: stimulus intensity, or threshold (% maximum stimulator output) required to produce the unconditioned test response (RMT); **channel 2**: sub-threshold conditioning stimulus (70% RMT); and **channel 3** tracks the stimulus required to produce target MEP when conditioned by a sub-threshold stimulus (70% RMT). Stimuli were delivered every 5 s and the computer advanced to next ISI only when tracking was stable.

SICI was measured as the increase in the test stimulus intensity required to evoke the target MEP. Inhibition was calculated off-line as follows [23]:
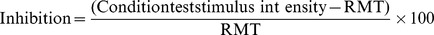



Facilitation was measured as the decrease in the conditioned test stimulus intensity required to evoke a target MEP.

**Single pulse TMS technique** was utilized to determine the MEP amplitude (mV), MEP onset latency (ms) and cortical silent period (CSP) duration (ms). The MEP amplitude was recorded with magnetic stimulus intensity set to 150% of RMT. Three stimuli were delivered at this level of stimulus intensity. Central motor conduction time (CMCT, ms) was calculated according to the F-wave method [25]. Cortical silent period duration was assessed by instructing the subject to contract the target muscle at ∼30% of maximal voluntary contraction with TMS intensity set to 150% of RMT. The CSP duration was measured from onset of MEP to return of EMG activity.

### Axonal Excitability

In the same sitting, axonal excitability studies were undertaken on the median and ulnar motor nerves according to a previously described protocol [26]. Compound muscle action potential (CMAP) responses were recorded from APB, FDI and ADM muscles with the active electrode positioned over the motor point and reference electrode placed over the base of the proximal thumb (APB and FDI) and fifth digit (ADM) respectively. From the CMAP amplitude, the split-hand index (SI) _ENREF_31was calculated according to the previously reported formula [6]:
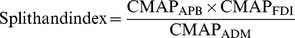



The following axonal excitability parameters were measured: (i) strength-duration time constant (τ_SD_) and rheobase; (ii) threshold electrotonus (TE) recorded with sub-threshold depolarizing currents at 10–20 ms (TEd [10–20 ms]), 40–60 ms (TEd [40–60 ms]), and 90–100 ms (TEd [90–100 ms]), and with hyperpolarizing currents at 10–20 ms, TEh [10–20 ms] and at 90–100 ms, TEh [90–100 ms]; (iii) hyperpolarizing current-threshold relationship (I/V) calculated from polarizing current between +50 and −100%; (iv) recovery cycle parameters including the relative refractory period (RRP, ms), superexcitability (%) and late subexcitability (%).

Recordings of the CMAP and MEP responses were amplified and filtered (3 Hz–3 kHz) using a Nikolet-Biomedical EA-2 amplifier (Cardinal Health Viking Select version 11.1.0, Viasys Healthcare Neurocare Group, Madison, Wisconsin, USA) and sampled at 10 kHz using a 16-bit data acquisition card (National Instruments PCI-MIO-16E-4). Responses were further filtered for electronic noise by using a Hum Bug (Hum Bug 50/60 Hz Noise Eliminator, Quest Scientific Instruments, North Vancouver, Canada). Data acquisition and stimulation delivery were controlled by QTRACS software (TROND-F, version 16/02/2009, © Professor Hugh Bostock, Institute of Neurology, Queen Square, London, UK). Temperature was monitored with a purpose built thermometer at the stimulation site.

### Statistical Analysis

Cortical and axonal excitability studies in ALS patients were compared to control data obtained from 21 healthy controls (13 men, 8 women: mean age 50 years, 24–67 years). Student t-test was used for assessing differences between two groups. Pearson's and Spearman's correlation coefficients were used to examine the relationship between parameters. Shapiro-Wilk test was used to assess for normality of data. A probability (P) value of <0.05 was considered statistically significant. Results were expressed as mean±standard error of the mean and median (interquartile range).

## Results

### Clinical features

At time of testing, the median disease duration was 9.5 months (6–17 months), indicating that those studies were undertaken towards the earlier stages of the disease (Table 1). In addition, the median ALSFRS-R score was 43 (41–46) while the median MRC sum score was 83 (79–88), suggesting a mild to moderate level of functional impairment at time of assessment. Seventy-three percent of patients exhibited limb-onset disease while 27% reported bulbar-onset disease.

**Table 1:** Clinical features for the 26 amyotrophic lateral sclerosis patients.

Patient	Age	Sex	Onset site	Duration (months)	ALSFRS-R	MRC Sum Score	SI
**1**	32	M	BULBAR	4	43	84	2.1
**2**	57	M	BULBAR	3	44	90	10
**3**	52	F	BULBAR	48	36	84	8.5
**4**	64	F	BULBAR	10	44	90	4.9
**5**	58	M	BULBAR	5	43	90	7.4
**6**	58	M	BULBAR	15	44	90	9.5
**7**	48	F	BULBAR	21	42	90	13
**8**	64	M	LIMB	7	47	87	5
**9**	69	M	LIMB	8	42	56	0
**10**	57	F	LIMB	9	45	83	6.4
**11**	64	M	LIMB	12	41	79	3.3
**12**	60	M	LIMB	6	42	82	1.7
**13**	62	M	LIMB	5	46	88	3.9
**14**	51	M	LIMB	20	33	65	0.1
**15**	42	M	LIMB	17	41	74	0
**16**	48	M	LIMB	5	42	80	9.8
**17**	56	F	LIMB	16	34	74	0.2
**18**	44	M	LIMB	3	46	86	0.9
**19**	58	F	LIMB	30	31	37	11.8
**20**	68	M	LIMB	10	48	88	6.3
**21**	69	F	LIMB	8	43	86	1.1
**22**	66	M	LIMB	9	47	80	2.8
**23**	69	F	LIMB	14	47	81	0
**24**	69	F	LIMB	8	46	79	7.9
**25**	73	M	LIMB	24	44	81	0
**26**	65	M	LIMB	17	41	71	5.3
**Mean**	**58.6**			**9.5**			**4.4**
**SEM**	**1.9**						**0.8**
**Median**					**43**	**83**	
**IQR**				**6**–**17**	**41**–**46**	**79**–**88**	

All patients were graded using the amyotrophic lateral sclerosis functional rating scale revised (ALSFRS-R). Muscle strength was assessed using the Medical Research Council (MRC) score. The split hand index (SI) was calculated in all patients.

The split-hand sign was evident in 62% of ALS patients at time of testing, but with follow-up 95% of patients developed a split-hand sign. The split-hand sign was more frequently observed in limb-onset ALS patients (73%) when compared to bulbar-onset disease (27%).

### Cortical excitability

Prior to undertaking cortical and axonal excitability studies, the degree of peripheral disease burden was formally assessed. There was a significant reduction of CMAP amplitude recorded over the APB (P<0.001), FDI (P<0.001) and ADM (P<0.001) muscles compared to controls. The split-hand index was significantly reduced in ALS (SI _ALS_ 4.7±0.8; SI _CONTROL_ 13.4±1.0, P<0.0005), confirming that the split-hand phenomenon was evident in the present ALS cohort.

**Figure 1:**
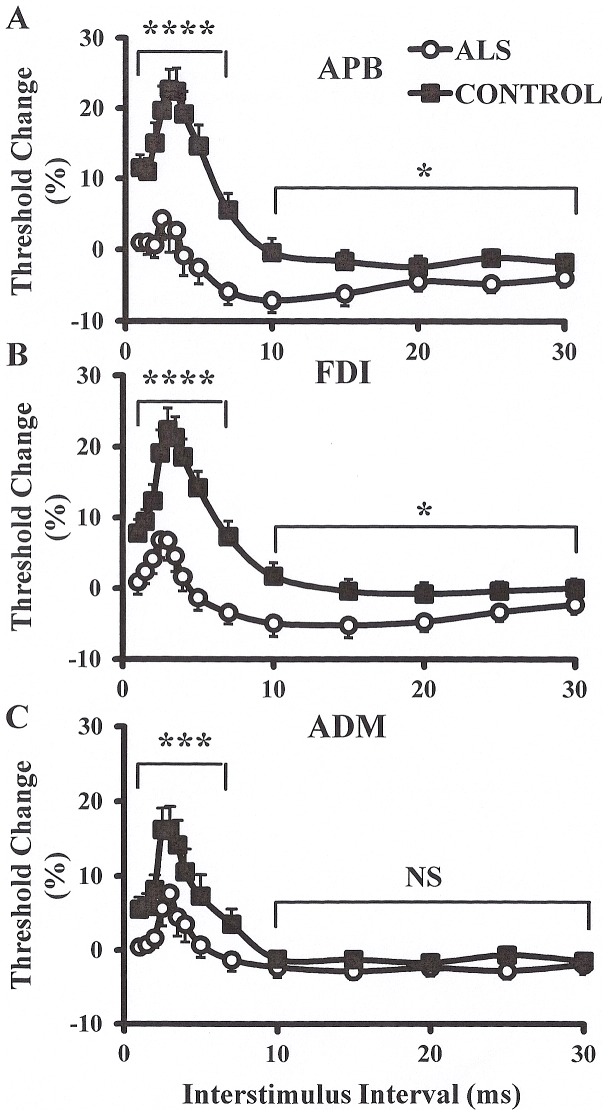
Short interval intracortical inhibition (SICI) and intracortical facilitation (ICF) are biomarkers of cortical function. SICI was significantly reduced and ICF increased when recording over the (A) abductor pollicis brevis (APB) and (B) first dorsal interosseous (FDI) muscles. (C) There was a significant reduction of SICI when recording over the abductor digit minimi (ADM) muscle, while there was no significant difference (NS) in ICF. The degree of SICI reduction was more prominent when recording over the APB and FDI muscles. *P<0.05; ***P<0.001; ****P<0.0001.

**Figure 2:**
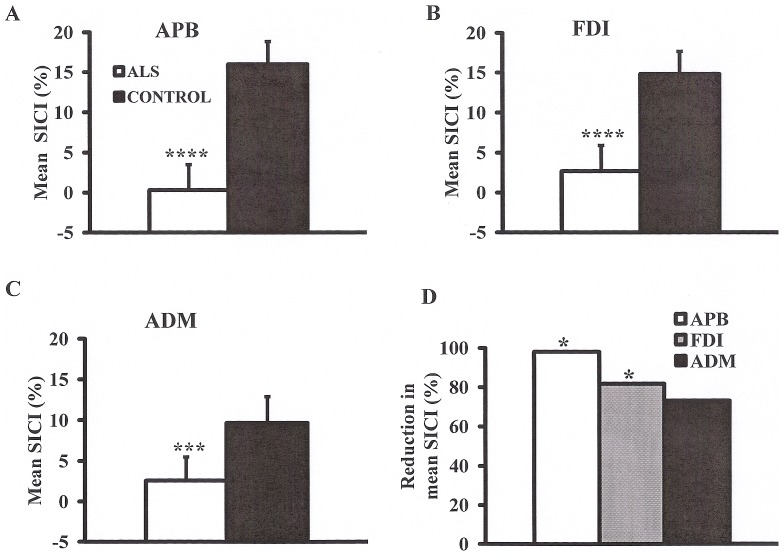
Mean short interval intracortical inhibition (SICI), over interstimulus intervals 1–7 ms, was significantly reduced when recording over the (**A**) abductor pollicis brevis (APB), (**B**) first dorsal interosseous (FDI), and (**C**) abductor digit minimi (ADM) muscles. (**D**) The reduction in mean SICI was most prominent when recording over the APB muscle. ***P<0.001; ****P<0.0001.

***Paired-pulse threshold tracking TMS*** studies disclosed a marked reduction of SICI across the range of intrinsic hand muscles, although this reduction was most prominent when recorded over the APB and FDI (Fig. 1A–C). Averaged SICI, between ISIs 1 to 7 ms, was significantly reduced over the APB (ALS 0.3±2.0%; controls 16.0±1.9%; P<0.0001), FDI (ALS 2.7±1.7%; controls 14.8±1.9%; P<0.0001) and ADM (ALS 2.6±1.5%; controls 9.7±2.2%; P<0.001, Fig. 2A–C) muscles, although the degree of reduction was more prominent over APB and FDI (SICI reduction _APB_, 98%; SICI reduction _FDI_ 81%; SICI reduction _ADM_ 73%, F = 2.8, P<0.05, Fig. 2D).

**Figure 3:**
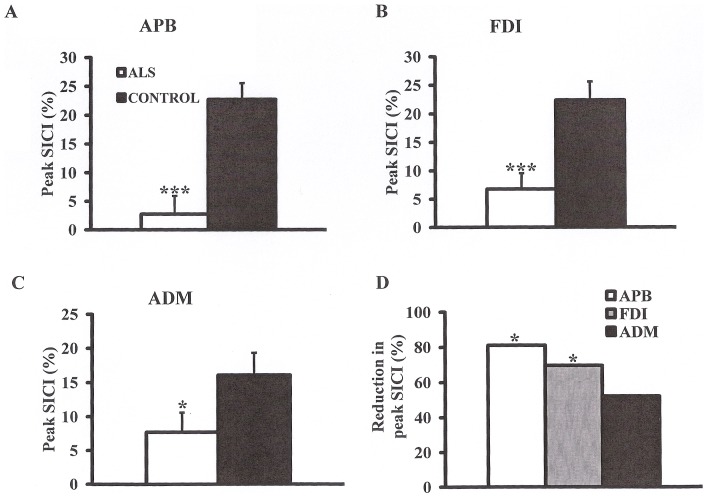
Short interval intracortical inhibition (SICI) peaks at interstimulus interval (ISI) of 3 ms. Peak SICI at interstimulus interval 3(**A**) abductor pollicis brevis (APB), (**B**) first dorsal interosseous (FDI), and (**C**) abductor digit minimi (ADM) muscles. (**D**) The reduction in peak SICI was most prominent when recording from the APB. *P<0.05; ***P<0.001.

Of further relevance, peak SICI at ISI 3 ms was also significantly reduced across the range of intrinsic hand muscles (ALS_APB_ 2.8±3.2%, controls _APB_ 22.7±2.8, P<0.001; ALS_FDI_ 6.8±2.8%, controls _FDI_ 23.5±2.5%, P<0.001; ALS_ADM_ 7.7±2.9%, controls _ADM_ 16.1±3.2%, P<0.05, Fig. 3A–C), although the reduction was again most prominent over the APB and FDI (SICI reduction _APB_, 81%; SICI reduction _FDI_ 70%; SICI reduction _ADM_ 52%, F = 2.0, P<0.05, Fig. 3D).

Following SICI, a period of **intracortical facilitation** develops between ISI 10–30 ms. The mean ICF between ISIs 10–30 ms was significantly increased in ALS patients when recorded over the APB (ALS −5.3±1.2%, controls −1.4±1.3%, P<0.05) and FDI muscles (ALS −4.1±1.4%, controls −0.53±1.4%, P<0.05) but not ADM (P = 0.18).

**Figure 4:**
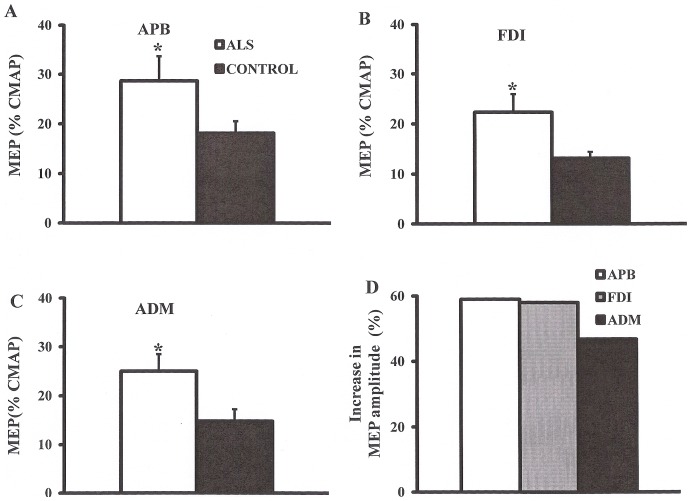
Motor evoked potential (MEP) amplitude, expressed as a percentage of the compound muscle action potential (CMAP) response, is a biomarker of corticomotoneuronal output. The MEP amplitude was significantly increased when recording over the (A) abductor pollicis brevis (APB), (B) first dorsal interosseous (FDI) and (C) abductor digit minimi (ADM) muscles. (D) The increase in MEP amplitude was most prominent when recording from the APB. *P<0.05; ***P<0.001.

***Single-pulse TMS technique*** disclosed that the MEP amplitude was significantly increased in ALS patients across the range of intrinsic hand muscles (ALS_APB_ 28.4±5.1%, controls _APB_ 18.1±2.4%, P<0.05; ALS_FDI_ 24.2±3.6%, controls _FDI_ 13.2±2.3%, P<0.05; ALS _ADM_ 22.9±2.9, controls _ADM_ 14.8±2.4%, P<0.05, Fig 4A–C). Of further relevance, there was a trend for the MEP amplitude increase to be greater when recording over the thenar muscles (APB _MEP INCREASE_ 59%; FDI _MEP INCREASE_ 57%; ADM _MEP INREASE_ 48.6%, Fig. 4D).

**Figure 5:**
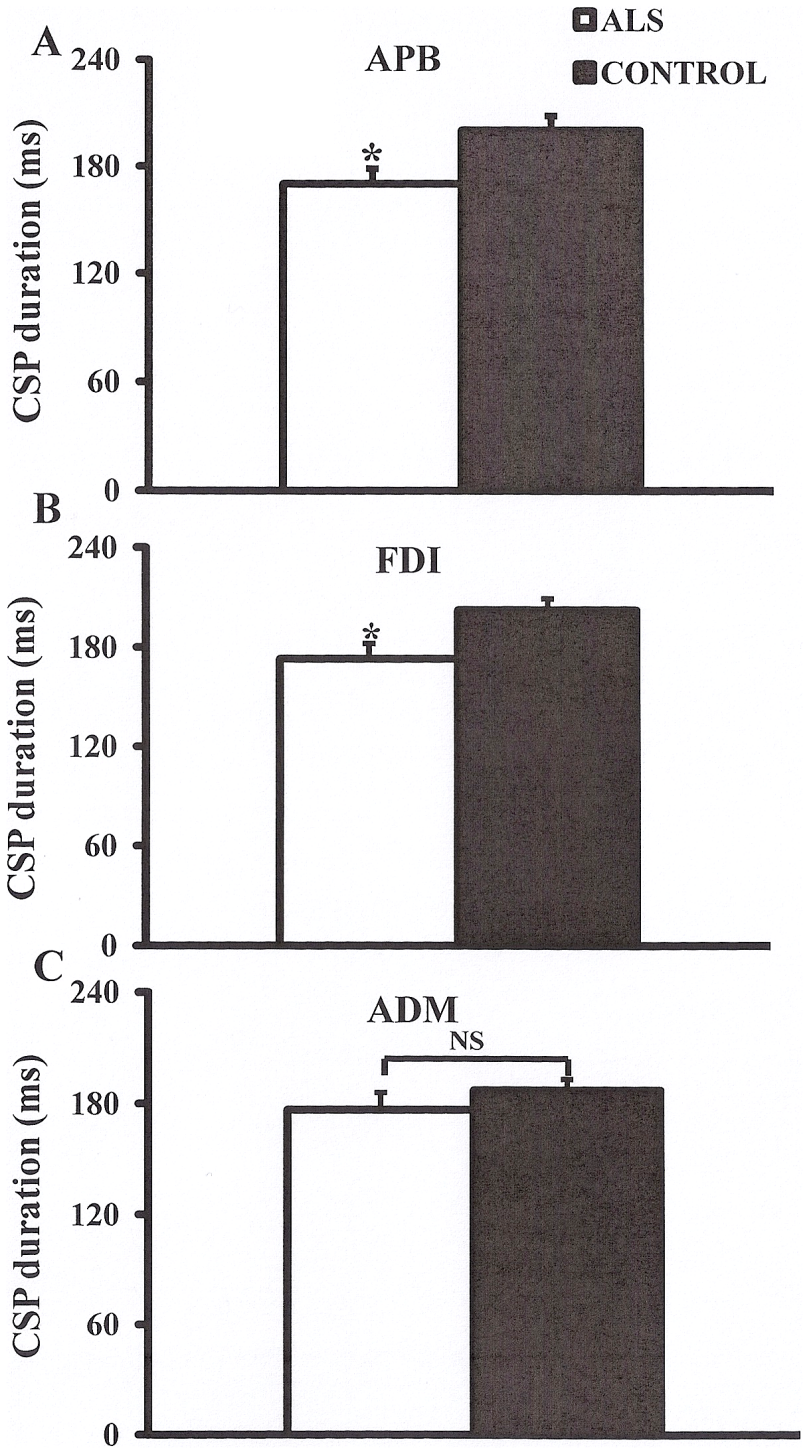
The cortical silent period (CSP) is a biomarker of cortical inhibitory circuit function. The CSP duration was significantly reduced when recording when recording over the (**A**) abductor pollicis brevis (APB) and (**B**) first dorsal interosseous (FDI) muscles. (**C**) There was no significant (NS) reduction in CSP duration when recording over the abductor digit minimi (ADM) muscle.

Of further relevance, the CSP duration was significantly reduced when recorded over the APB (ALS 170.1±8.5 ms, controls 200.4±8.1 ms, P<0.05) and FDI (ALS 173.1±9.2 ms, controls 202.4±6.8 ms, P<0.05) but not ADM (ALS 176.8±9.2 ms, controls 187.2±6.0 ms, P = 0.17, Fig. 5). In contrast, there were no significant differences in RMT (ALS _APB_ 56.1±1.9%, controls _APB_ 56.2±1.8%, P = 0.48; ALS _FDI_ 55.5±1.9%, controls _FDI_ 58.0±2.1, P = 0.20; ALS _ADM_ 54.5±1.8, controls _ADM_ 56.3±2.0, P = 0.25) and central motor conduction time (F = 0.01, P = 0.99) between groups.

### Axonal excitability

**Figure 6:**
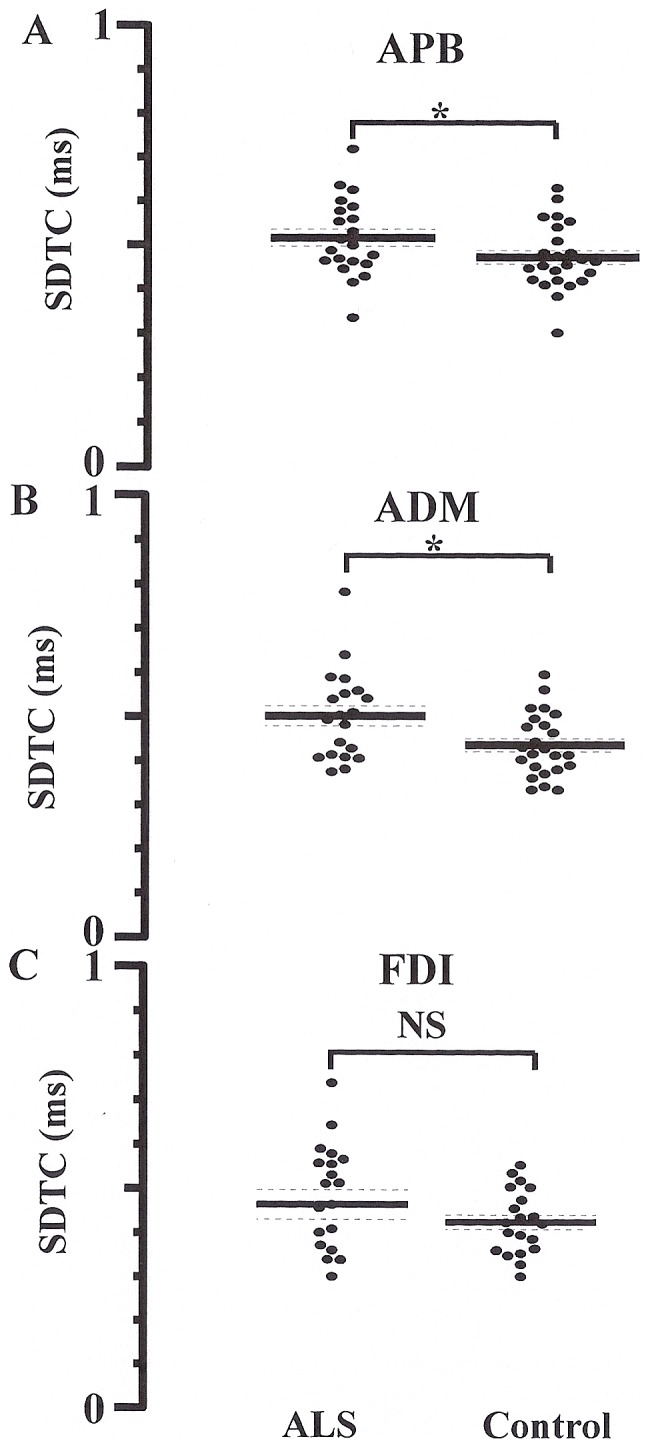
Strength duration time constant (SDTC) is a biomarker of persistent Na^+^ conductances. The SDTC was significantly increased when recording from the (**A**) abductor pollicis brevis (APB) and (**B**) abductor digit minim (ADM) muscles, but not (**C**) first dorsal interosseous (FDI). *P<0.05.

The strength-duration time constant, a biomarker of nodal persistent Na^+^ conductance [27], [28], [29], [30], [31], was significantly increased when recorded over APB (ALS 0.51±0.02 ms, controls 0.46±0.02 ms, P<0.05, Fig. 6A) and ADM (ALS 0.49±0.02 ms, controls 0.44±0.02 ms, P<0.05, Fig. 6B) muscles but not FDI (ALS 0.47±0.02 ms, controls 0.42±0.02 ms, P = 0.06, Fig 6C). In contrast, there were no significant differences in rheobase between ALS patients and controls across the range of intrinsic hand muscles (APB, P = 0.19; FDI, P = 0.49; ADM, P = 0.33).

***Threshold Electrotonus***, a biomarker of internodal and paranodal K^+^ conductance [30], again disclosed the presence of the type I abnormality of TE, whereby sub-threshold depolarizing currents induced greater changes in threshold [32] when recording from all three muscles. Specifically, depolarizing TE at 90–100 ms was significantly greater in ALS patients when recorded from the APB (ALS 50.3±1.5%; controls 44.7±0.8%, P<0.001), ADM (ALS 48.5±1.0%; controls 45.5±0.7%, P<0.05) and FDI (ALS 45.1±1.5%; controls 40.0±2.2%, P<0.05) muscles. These changes in depolarising TE were accompanied by a significant increase in TEd at 40–60 ms when recording over APB (ALS 56.1±1.7%; controls 52.6±1.0%, P<0.05) and ADM (ALS 53.6±1.3%; controls 50.8±0.9%, P<0.05), but not FDI motor axons (P = 0.11)

Of further relevance, superexcitability was significantly increased when recording from the APB (ALS −25.9±1.4%; controls −21.6±1.5%, P<0.05) and FDI (ALS −27.3±1.4%; controls −23.1±1.2%, P<0.05), but not ADM (ALS −25.1±1.2%; controls −23.5±1.3%, P = 0.19). In contrast, there were no significant changes in the relative refractory period (APB, P = 0.18; FDI, P = 0.14; ADM, P = 0.37) and late subexcitability (APB, P = 0.12; FDI, P = 0.15; ADM, P = 0.10) between ALS patients and controls across the range of intrinsic hand muscles. In addition, hyperpolarising I/V gradient was significantly increased in ALS patients when recording from the APB muscle (ALS 0.41±0.02, controls 0.36±0.01, P<0.05), but not the FDI (P = 0.08) and ADM (P = 0.12). Taken together, these findings reveal that while the abnormalities of axonal excitability were evident in ALS, they did not appear to follow a split-hand pattern.

### Correlation studies

Combining clinical parameters with measures of cortical and axonal excitability, it was evident that the MEP amplitude (R = −0.40, P<0.01) and CSP duration (R = 0.34, P<0.01) were significantly correlated with the split-hand index. Of relevance, there was a significant correlation between SICI and MRC upper limb score (R = −0.3, P<0.05). In contrast, there was no significant correlation between any of the axonal excitability parameters and split-hand index, CMAP amplitude, measures of cortical excitability and clinical parameters. Taken together, these findings suggest that cortical hyperexcitability may be linked to the development of the split-hand phenomenon in ALS.

## Discussion

In the present study, cortical and peripheral axonal techniques were utilised to dissect the relative contribution of central and peripheral processes in development of the ALS split-hand. Cortical hyperexcitability, as indicated by a significant reduction in short interval intracortical inhibition and cortical silent period duration along with increases in motor evoked potential amplitude, was evident across the range of intrinsic hand muscles, although the degree of cortical hyperexcitability was most prominent when recorded from the APB and FDI muscles. Of further relevance, there was a significant correlation between measures of cortical excitability and the split-hand index, suggesting that cortical hyperexcitability was associated with development of the split-hand phenomenon in ALS. At a peripheral level, upregulation of persistent Na^+^ conductances along with reduction in K^+^ currents was evident, but did not appear to follow a split-hand distribution and was not correlated with the split-hand index, thereby arguing against a significant peripheral contribution in driving the development of the split-hand phenomenon in ALS. The mechanisms underlying these central and peripheral excitability changes and their relevance for development of the split-hand phenomenon, with implications for ALS pathophysiology, will be discussed.

### Origins of the split-hand phenomenon in ALS

While the pathophysiological mechanisms underlying the development of the split-hand phenomenon in ALS have not been established, central and peripheral processes have been implicated [4]. A cortical basis was inferred from clinical observations that thenar muscles (APB/FDI) were critical in execution of complex hand tasks [4], and thereby would exhibit a greater cortical representation, a notion supported by TMS studies in healthy controls [14], [16], [33]. Importantly preferential dysfunction of corticomotoneuronal pathways was previously established in ALS [16].

The novel findings from the present study provide critical support for a cortical basis in development of the ALS split-hand. Specifically, cortical dysfunction was heralded by marked reduction of SICI, a biomarker of strong inhibitory intracortical GABAergic function and weaker cortical glutaminergic facilitatory effects [34]. Importantly, abnormalities of SICI in ALS appear to be mediated by a combination of glutamate excitotoxicity and degeneration of inhibitory cortical neurons[35], [36]_ENREF_53. The findings of more prominent SICI reduction when recorded over APB and FDI, would imply a greater level of cortical hyperexcitability to the APB and FDI motor neurons. Of further relevance, intracortical facilitation, a biomarker of glutaminergic function [34], [37], was significantly increased when recorded from the APB and FDI but not hypothenar muscles. Taken together, these findings suggest that cortical processes, namely cortical hyperexcitability, may underlie the development of the split-hand sign in ALS.

There was also a marked increase in MEP amplitude in the ALS cohort and was correlated with the split-hand index. Given that the MEP amplitude reflects the density of corticomotoneuronal projections onto motor neurons [38] _ENREF_31 as well as the level of glutamatergic neurotransmission in the central nervous system [37], the present findings lend further credence to the notion that cortical processes contribute to the development of the split-hand sign in ALS. Alternatively, it could also be argued that the increase in MEP amplitude may represent less phase cancellation due to fewer motor units or possibly repetitive firing of corticomotoneurons [39]. In addition to changes in MEP amplitudes, a significant reduction of CSP duration was evident in the ALS cohort, but only when recorded over the thenar muscles (APB/FDI). Importantly, the CSP duration reflects the degree of cortical inhibition and appears to be mediated by inhibitory neurons acting via GABA_B_ receptors [40], [41], [42], [43], [44], [45]. Consequently, findings that identified a significant reduction in CSP duration when recording over APB/FDI muscles, suggest a greater level of cortical disinhibition and cortical hyperexcitability to the APB/FDI muscles, providing further support for a cortical mechanism as the basis of the split-hand phenomenon in ALS.

Alternatively, it could be argued that the differences in cortical excitability between the intrinsic muscles could be related to an interaction between stimulated cortical areas. Given that electromyography techniques were not utilised to ensure electrical silence of the “non-stimulated” intrinsic hand muscles, especially the APB and FDI, such a notion could not be absolutely discounted.

It has also been argued that peripheral mechanisms may contribute to development of the split-hand sign in ALS [4]. More prominent membrane excitability abnormalities in the APB motor neurons, as indicated by longer τ_SD_, was recently reported in ALS [17]. Given that τ_SD_ is a biomarker of persistent Na^+^ conductances [30], and linked to axonal degeneration [10], [46], [47], these findings implied that upregulation of persistent Na^+^ conductances contributed to development of the split-hand phenomenon, although the properties of FDI motor axons were not assessed. In contrast, the present study established a comparable increase of τ_SD_ in APB and ADM axons, without significant increases of τ_SD_ in FDI axons, thereby arguing against a significant contribution of axonal dysfunction in development of the split-hand phenomenon in ALS.

In addition, a significant reduction in depolarising TE was also evident across the range of recorded motor axons. Given that depolarising TE is a biomarker of slow K^+^ currents [30], the findings in the present study suggest that reduced slow K^+^ currents is a feature of ALS and in keeping with previous studies [18], [46], [48]. Importantly, the changes in depolarising TE did not appear to follow a split-hand distribution, thereby further arguing against a significant contribution of peripheral processes in development of the split-hand phenomenon in ALS.

### Split-hand phenomenon and ALS pathophysiology

Emerging evidence suggests that genetic factors and molecular processes underlie the development of ALS [1]. Cortical hyperexcitability was proposed as an important pathophysiological mechanism, whereby motor neuron degeneration was mediated via glutamate excitotoxicity process [7]. Support for such a framework has been provided by TMS studies [9], [11] as well as transgenic SOD-1 mouse models studies [49]. Additional support for glutamate-mediated excitotoxity is provided by transgenic SOD-1 mouse model and human studies identifying abnormalities of the astrocytic glutamate transporter, excitatory amino acid transporter-2 [50], [51]. Importantly, ALS motor neurons exhibit increased expression of Ca^2+^-permeable AMPA receptors, thereby rendering these more susceptible to excitotoxicity [52].

The findings from the present study of more prominent cortical hyperexcitability to the APB and FDI muscles, together with an absence of a split-hand distribution of axonal excitability abnormalities, provide support for a cortical basis to ALS pathogenesis. The significant correlation between cortical hyperexcitability and the split-hand index, further suggests that glutamate-mediated cortical hyperexcitability may underlie the preferential degeneration of motor neurons in ALS.

## References

[1] Kiernan MC, Vucic S, Cheah BC, Turner MR, Eisen A, et al (2011) Amyotrophic lateral sclerosis. Lancet 377: 942–955. 21296405 10.1016/S0140-6736(10)61156-7

[2] Wilbourn AJ (2000) The “split hand syndrome”. Muscle Nerve 23: 138. 10.1002/(sici)1097-4598(200001)23:1<138::aid-mus22>3.0.co;2-7 10590421

[3] Kuwabara S, Sonoo M, Komori T, Shimizu T, Hirashima F, et al (2008) Dissociated small hand muscle atrophy in amyotrophic lateral sclerosis: frequency, extent, and specificity. Muscle Nerve 37: 426–430. 18236469 10.1002/mus.20949

[4] Eisen A, Kuwabara S (2012) The split hand syndrome in amyotrophic lateral sclerosis. J Neurol, Neurosurg Psychiatry 83: 399–403. 22100761 10.1136/jnnp-2011-301456

[5] Bae JS, Sawai S, Misawa S, Kanai K, Isose S, et al (2009) Differences in excitability properties of FDI and ADM motor axons. Muscle Nerve 39: 350–354. 19208410 10.1002/mus.21107

[6] Menon P, Kiernan MC, Yiannikas C, Stroud J, Vucic S (2013) Split-hand index for the diagnosis of amyotrophic lateral sclerosis. Clin Neurophysiol 124: 410–416. 23017503 10.1016/j.clinph.2012.07.025

[7] Eisen A, Kim S, Pant B (1992) Amyotrophic lateral sclerosis (ALS): a phylogenetic disease of the corticomotoneuron? Muscle Nerve 15: 219–224. 1549143 10.1002/mus.880150215

[8] Prout AJ, Eisen A (1994) The cortical silent period and ALS. Muscle Nerve 17: 217–223. 8114792 10.1002/mus.880170213

[9] Vucic S, Kiernan MC (2006) Novel threshold tracking techniques suggest that cortical hyperexcitability is an early feature of motor neuron disease. Brain 129: 2436–2446. 16835248 10.1093/brain/awl172

[10] Vucic S, Kiernan MC (2010) Upregulation of persistent sodium conductances in familial ALS. J Neurol Neurosurg Psychiatry 81: 222–227. 19726402 10.1136/jnnp.2009.183079

[11] Vucic S, Nicholson GA, Kiernan MC (2008) Cortical hyperexcitability may precede the onset of familial amyotrophic lateral sclerosis. Brain 131: 1540–1550. 18469020 10.1093/brain/awn071

[12] Vucic S, Cheah BC, Yiannikas C, Kiernan MC (2011) Cortical excitability distinguishes ALS from mimic disorders. Clin Neurophysiol 122: 1860–1866. 21382747 10.1016/j.clinph.2010.12.062

[13] Lemon RN, Griffiths J (2005) Comparing the function of the corticospinal system in different species: organizational differences for motor specialization? Muscle Nerve 32: 261–279. 15806550 10.1002/mus.20333

[14] Abbruzzese G, Assini A, Buccolieri A, Schieppati M, Trompetto C (1999) Comparison of intracortical inhibition and facilitation in distal and proximal arm muscles in humans. J Physiol (Lond) 514: 895–903. 9882759 10.1111/j.1469-7793.1999.895ad.x PMC2269103

[15] Friedman AP, Freedman D (1950) Amyotrophic lateral sclerosis. The Journal of nervous and mental disease 111: 1–18. 15402614 10.1097/00005053-195011110-00001

[16] Weber M, Eisen A, Stewart H, Hirota N (2000) The split hand in ALS has a cortical basis. J Neurol Sci 180: 66–70. 11090867 10.1016/s0022-510x(00)00430-5

[17] Shibuya K, Misawa S, Nasu S, Sekiguchi Y, Mitsuma S, et al (2013) Split hand syndrome in amyotrophic lateral sclerosis: different excitability changes in the thenar and hypothenar motor axons. J Neurol Neurosurg Psychiatry 84: 969–972. 23467416 10.1136/jnnp-2012-304109

[18] Rowland LP, Shneider NA (2001) Amyotrophic lateral sclerosis. New England Journal of Medicine 344: 1688–1700. 11386269 10.1056/NEJM200105313442207

[19] de Carvalho M, Dengler R, Eisen A, England JD, Kaji R, et al (2008) Electrodiagnostic criteria for diagnosis of ALS. Clin Neurophysiol 119: 497–503. 18164242 10.1016/j.clinph.2007.09.143

[20] Cedarbaum JM, Stambler N, Malta E, Fuller C, Hilt D, et al (1999) The ALSFRS-R: a revised ALS functional rating scale that incorporates assessments of respiratory function. BDNF ALS Study Group (Phase III). J Neurol Sci 169: 13–21. 10540002 10.1016/s0022-510x(99)00210-5

[21] O'Brien MD (2004) Aid to the examination of the peripheral nervous system. 4 ed. London: W.B.Saunders. pp. 1–3.

[22] Fisher RJ, Nakamura Y, Bestmann S, Rothwell JC, Bostock H (2002) Two phases of intracortical inhibition revealed by transcranial magnetic threshold tracking. Exp Brain Res 143: 240–248. 11880900 10.1007/s00221-001-0988-2

[23] Vucic S, Howells J, Trevillion L, Kiernan MC (2006) Assessment of cortical excitability using threshold tracking techniques. Muscle Nerve 33: 477–486. 16315324 10.1002/mus.20481

[24] Menon P, Kiernan MC, Vucic S (2013) Cortical excitability differences in hand muscles follow a split-hand pattern in healthy controls. Muscle Nerve: doi: 10.1002/mus.24072. 10.1002/mus.24072 24037729

[25] Mills KR, Murray NM (1986) Electrical stimulation over the human vertebral column: which neural elements are excited? Electroencephalogr Clin Neurophysiol 63: 582–589. 2422007 10.1016/0013-4694(86)90145-8

[26] Kiernan MC, Burke D, Andersen KV, Bostock H (2000) Multiple measures of axonal excitability: a new approach in clinical testing. Muscle Nerve 23: 399–409. 10679717 10.1002/(sici)1097-4598(200003)23:3<399::aid-mus12>3.0.co;2-g

[27] Mogyoros I, Kiernan MC, Burke D (1996) Strength-duration properties of human peripheral nerve. Brain 119: 439–447. 8800939 10.1093/brain/119.2.439

[28] Bostock H (1983) The strength-duration relationship for excitation of myelinated nerve: computed dependence on membrane parameters. J Physiol (Lond) 341: 59–74. 6312032 10.1113/jphysiol.1983.sp014792 PMC1195322

[29] Bostock H, Cikurel K, Burke D (1998) Threshold tracking techniques in the study of human peripheral nerve. Muscle Nerve 21: 137–158. 9466589 10.1002/(sici)1097-4598(199802)21:2<137::aid-mus1>3.0.co;2-c

[30] Burke D, Kiernan MC, Bostock H (2001) Excitability of human axons. Clin Neurophysiol 112: 1575–1585. 11514239 10.1016/s1388-2457(01)00595-8

[31] Bostock H, Rothwell JC (1997) Latent addition in motor and sensory fibres of human peripheral nerve. J Physiol (Lond) 498: 277–294. 9023784 10.1113/jphysiol.1997.sp021857 PMC1159250

[32] Bostock H (1995) Mechanisms of accommodation and adaptation in myelinated axons. In: Waxman SG, Kocsis JD, Stys PK, editors. The Axon. New York: Oxford Univerisy Press. pp. 311–327.

[33] Bae JS, Menon P, Mioshi E, Kiernan MC, Vucic S (2013) Cortical excitability differences between flexor pollicis longus and APB. Neurosci Lett 541: 150–154. 23499957 10.1016/j.neulet.2013.03.003

[34] Ziemann U (2004) TMS and drugs. Clin Neurophysiol 115: 1717–1729. 15261850 10.1016/j.clinph.2004.03.006

[35] Vucic S, Lin CS-Y, Cheah BC, Murray J, Menon P, et al (2013) Riluzole exerts central and peripheral modulating effects in amyotrophic lateral sclerosis. Brain 136: 1361–1370. 23616585 10.1093/brain/awt085

[36] Nihei K, McKee AC, Kowall NW (1993) Patterns of neuronal degeneration in the motor cortex of amyotrophic lateral sclerosis patients. Acta Neuropathologica 86: 55–64. 8396837 10.1007/BF00454899

[37] Vucic S, Ziemann U, Eisen A, Hallett M, Kiernan MC (2013) Transcranial magnetic stimulation and amyotrophic lateral sclerosis: pathophysiological insights. J Neurol Neurosurg Psychiatry 84: 1161–1170. 23264687 10.1136/jnnp-2012-304019 PMC3786661

[38] Chen R, Cros D, Curra A, Di Lazzaro V, Lefaucheur JP, et al (2008) The clinical diagnostic utility of transcranial magnetic stimulation: report of an IFCN committee. Clin Neurophysiol 119: 504–532. 18063409 10.1016/j.clinph.2007.10.014

[39] Komissarow L, Rollnik JD, Bogdanova D, Krampfl K, Khabirov FA, et al (2004) Triple stimulation technique (TST) in amyotrophic lateral sclerosis.[see comment]. Clinical Neurophysiology 115: 356–360. 14744577 10.1016/j.clinph.2003.10.003

[40] Cantello R, Gianelli M, Civardi C, Mutani R (1992) Magnetic brain stimulation: the silent period after the motor evoked potential. Neurology 42: 1951–1959. 1407578 10.1212/wnl.42.10.1951

[41] Chen R, Lozano AM, Ashby P (1999) Mechanism of the silent period following transcranial magnetic stimulation. Evidence from epidural recordings. Exp Brain Res 128: 539–542. 10541749 10.1007/s002210050878

[42] Inghilleri M, Berardelli A, Cruccu G, Manfredi M (1993) Silent period evoked by transcranial stimulation of the human cortex and cervicomedullary junction. J Physiol (Lond) 466: 521–534. 8410704 PMC1175490

[43] Siebner HR, Dressnandt J, Auer C, Conrad B (1998) Continuous intrathecal baclofen infusions induced a marked increase of the transcranially evoked silent period in a patient with generalized dystonia. Muscle Nerve 21: 1209–1212. 9703450 10.1002/(sici)1097-4598(199809)21:9<1209::aid-mus15>3.0.co;2-m

[44] Werhahn KJ, Kunesch E, Noachtar S, Benecke R, Classen J (1999) Differential effects on motorcortical inhibition induced by blockade of GABA uptake in humans. J Physiol (Lond) 517: 591–597. 10332104 10.1111/j.1469-7793.1999.0591t.x PMC2269337

[45] Stetkarova I, Kofler M (2012) Differential effects of cortical and spinal inhibitory circuits. Clin Neurophysiol: In press. 10.1016/j.clinph.2012.07.005 22877625

[46] Kanai K, Kuwabara S, Misawa S, Tamura N, Ogawara K, et al (2006) Altered axonal excitability properties in amyotrophic lateral sclerosis: impaired potassium channel function related to disease stage. Brain 129: 953–962. 16467388 10.1093/brain/awl024

[47] Kuo JJ, Siddique T, Fu R, Heckman CJ (2005) Increased persistent Na(+) current and its effect on excitability in motoneurones cultured from mutant SOD1 mice. J Physiol (Lond) 563: 843–854. 15649979 10.1113/jphysiol.2004.074138 PMC1665614

[48] Vucic S, Kiernan MC (2006) Axonal excitability properties in amyotrophic lateral sclerosis. Clin Neurophysiol 117: 1458–1466. 16759905 10.1016/j.clinph.2006.04.016

[49] Browne SE, Yang L, DiMauro JP, Fuller SW, Licata SC, et al (2006) Bioenergetic abnormalities in discrete cerebral motor pathways presage spinal cord pathology in the G93A SOD1 mouse model of ALS. Neurobiol Dis 22: 599–610. 16616851 10.1016/j.nbd.2006.01.001

[50] Trotti D, Rolfs A, Danbolt N, Brown R, Hediger M (1999) SOD1 mutants linked to amyotrophic lateral sclerosis selectively inactivate a glial glutamate transporter. Nat Neurosci 2: 427–433. 10321246 10.1038/8091

[51] Rothstein JD, Patel S, Regan MR, Haenggeli C, Huang YH, et al (2005) Beta-lactam antibiotics offer neuroprotection by increasing glutamate transporter expression. Nature 433: 73–77. 15635412 10.1038/nature03180

[52] Van Damme P, Braeken D, Callewaert G, Robberecht W, Van Den Bosch L (2005) GluR2 deficiency accelerates motor neuron degeneration in a mouse model of amyotrophic lateral sclerosis. J Neuropathol Exp Neurol 64: 605–612. 16042312 10.1097/01.jnen.0000171647.09589.07

